# Expression of miR-142-5p in Peripheral Blood Mononuclear Cells from Renal Transplant Patients with Chronic Antibody-Mediated Rejection

**DOI:** 10.1371/journal.pone.0060702

**Published:** 2013-04-05

**Authors:** Richard Danger, Chloé Paul, Magali Giral, Amélie Lavault, Yohann Foucher, Nicolas Degauque, Annaïck Pallier, Maxim Durand, Stéphanie Castagnet, Jean-Paul Duong Van Huyen, Michel Delahousse, Karine Renaudin, Jean-Paul Soulillou, Sophie Brouard

**Affiliations:** 1 Institut National de la Santé Et de la Recherche Médicale INSERM U1064 and Institut de Transplantation Urologie Néphrologie (ITUN), Nantes, France; 2 Faculté de médecine, Université de Nantes, Nantes, France; 3 Centre Hospitalier Universitaire, Hôtel Dieu, Nantes, France; 4 Service d'Anatomie Pathologique, Hôpital Européen Georges Pompidou, Paris, France; 5 Service de Néphrologie et Transplantation Rénale, Hôpital Foch, Suresnes, France; IPMC, CNRS UMR 7275 UNS, France

## Abstract

In renal transplantation, the unresponsiveness of patients undergoing chronic antibody mediated rejection (CAMR) to classical treatment stress on the need for accurate biomarkers to improve its diagnosis. We aim to determine whether microRNA expression patterns may be associated with a diagnosis of CAMR. We performed expression profiling of miRNAs in peripheral blood mononuclear cells (PBMC) of kidney transplant recipients with CAMR or stable graft function. Among 257 expressed miRNAs, 10 miRNAs associated with CAMR were selected. Among them, miR-142-5p was increased in PBMC and biopsies of patients with CAMR as well as in a rodent model of CAMR. The lack of modulation of miR-142-5p in PBMC of patients with renal failure, suggests that its over-expression in CAMR was associated with immunological disorders rather than renal dysfunction. A ROC curve analysis performed on independent samples showed that miR-142-5p is a potential biomarker of CAMR allowing a very good discrimination of the patients with CAMR (AUC = 0.74; p = 0.0056). Moreover, its expression was decreased in PHA-activated blood cells and was not modulated in PBMC from patients with acute rejection, excluding a non-specific T cell activation expression. The absence of modulation of this miRNA in immunosuppressed patients suggests that its expression was not influenced by treatment. Finally, the analysis of miR-142-5p predicted targets under-expressed in CAMR PBMC in a published microarray dataset revealed an enrichment of immune-related genes. Altogether, these data suggest that miR-142-5p could be used as a biomarker in CAMR and these finding may improve our understanding of chronic rejection mechanisms.

## Introduction

Chronic antibody-mediated rejection (CAMR) is a major cause of kidney graft loss after one year [1]. The process leading to this phenomenon is not yet fully understood [2], [3] Furthermore, whereas the diagnosis of CAMR is established by histological analysis and detection of circulating Donor Specifc Antibodies (DSA) [4], predicting its future occurrence remains elusive and functional parameters such as creatinemia and proteinuria, currently used in clinical practice, cannot detect CAMR early enough to prevent irreversible graft alterations. In addition, despite being highly specific, C4d deposits display a now well-recognized lack of sensitivity and the presence of anti-HLA antibodies or DSA can be associated with normal graft function for years [1], [5]. Thus, the identification of early molecular markers of CAMR would be beneficial, in order to adjust treatment to prevent and limit graft injury. There is currently growing interest in microRNAs (miRNAs), which can repress the expression of numerous genes and thereby influence large downstream networks [6]. These small molecules are involved in various biological mechanisms and diseases as well as in the regulation of immune mechanisms. miRNAs have been reported in renal transplantation as modulating gene expression in biopsies and/or blood from recipients undergoing acute cellular rejection [7]–[9], fibrosis [10], [11] or operational tolerance [12]. But to date, whereas the high stability of miRNAs favors them as potential biomarkers [13], their detection and potential role in CAMR have not yet been explored.

In this study, we performed profiling of miRNAs expressed in patients with CAMR compared to patients with stable graft function (STA). Among them, we focused on miR-142-5p, which was over-expressed in peripheral blood mononuclear cells (PBMC) and biopsies of CAMR patients, as well as in a rodent model of CAMR, and could therefore be used as a diagnostic biomarker in CAMR. Finally, we reported though a database analysis of molecular target that CAMR harbored a clear fingerprint of miR-142-5p target genes.

## Materials and Methods

### Patients

The study was approved by the University Hospital Ethical Committee and the Committee for the Protection of Patients from Biological Risks. All participating patients gave written informed consent. Histology was classified according to pathologists and the updated 2009 Banff classification criteria [4]. A total of 112 patients and 11 healthy volunteers were included for study and detailed clinical data are presented in Table S1.

Blood and biopsy samples were obtained from patients with the following status at the time of blood collection: *i* ) Kidney recipients with stable graft function (STA; n = 53): these patients had stable renal function as estimated by a senior clinician, with stable creatinemia (±25% of the mean value of creatinemia in the year preceding inclusion) (mean = 112.8±3.96 µmol/L (±Standard Error)) and a daily proteinuria less than 1 g/24 h (mean = 0.16±0.19 g/24 h). miRNA expression was analyzed in 18 biopsies that exhibited normal histological features (excepted 2 interstitial fibrosis/tubular atrophy grade I and 3 calcineurin-inhibitor toxicity). For the remaining patients, and in order to avoid the inclusion of infraclinical rejection -and because biopsy would not be performed in patients with very stable graft function for ethical reasons-, we surveyed the graft function of these patients after at least one year of inclusion. According to our criteria, all the STA patients remained stable at this point; *ii* ) Patients with chronic antibody-mediated rejection (CAMR) (n = 40): These patients were classified as having CAMR based on renal dysfunction and histology. Renal dysfunction was determined by a slow and progressive increase of creatinemia over months (mean = 247.1±25.53 µmol/L) and/or a daily proteinuria more than 1 g/24 h (mean = 2.30±0.39 g/24 h). CAMR was biopsy proven according to the 2009 update of the Banff classification [4] that was defined by histological evidences of chronic tissue injury (*i.e.* allograft glomerulopathy (cg>0) and/or interstitial fibrosis/tubular atrophy (ci+ct>0) and/or fibrous intimal thickening in arteries (cv>0)) associated with the presence of C4d and DSA (if one of the 2 last criteria is lacking, the diagnosis is considered “suspicious” CAMR (sCAMR)); *iii* ) patients with renal failure (RF; n = 10): These patients displayed kidney dysfunction of non-immunological cause and had not received a transplant, immunosuppressant drugs or dialysis (mean creatinemia = 525±57.9 µmol/L); *iv* ) Patients with biopsy-proven acute rejection (AR) (n = 9): These patients exhibited a rapid increase in creatinemia or proteinuria (over days) (mean creatinemia = 333.0±58.36 µmol/L and mean proteinuria = 1.69±0.68 g/24 h) and active acute rejection as demonstrated by histology according to the 2009 update Banff classification [4] at the time of blood sampling. *v* ) Healthy volunteers (HV) (n = 11): had a normal blood formula and no infectious or other concomitant pathology for at least 6 months before the study.

### Sample preparation

Renal graft biopsies were collected using 18-G disposable needles, immediately snap-frozen in liquid nitrogen and stored at −80°C. Venous blood samples were collected in EDTA vacutainers and processed for analysis within 4 hours. Peripheral Blood Mononuclear Cells (PBMC) were separated on a Ficoll layer (Eurobio, Les Ulis, France). Total RNA was extracted using the TRIzol® method (Invitrogen, Cergy Pontoise, France) according to the manufacturer's instructions.

### Heart transplant model of CAMR

Hearts from LEW.1W donors were transplanted into MHC-mismatched LEW.1A recipients receiving a donor-specific blood transfusion (DST) 7 and 14 days before transplantation [14], [15]. The graft from the DST-treated recipients were studied at days 100 post transplantation when the grafts exhibit histological signs of CAMR as previously reported [14].

### microRNA profiling

miRNA profiling was performed using TaqMan Arrays MicroRNA Cards pool A set v2.0 (Applied Biosystems, Foster City, CA, USA) with pre-amplification and following the manufacturer's recommendations, on an ABI Prism 7900 HT (Applied Biosystems). miRNAs with more than half of the cycle of quantification (Cq) values above 35 per group were eliminated. For each sample, normalization was performed by subtraction of the median measured Cq, resulting in the ΔCq value [16]. In order to identify potential differentially expressed miRNAs, miRNAs were ranked using p-values from non-parametric Mann-Whitney tests, which does not require normal assumptions and asymptotic conditions. These p-values are uncorrected for multiple testing and thus not an absolute identification of significant differential miRNAs, further validation is required for each miRNA.

### microRNA individual assays

Individual microRNA expression was measured with the TaqMan miRNA assay protocol (Applied Biosystems, Foster City, CA, USA) using probes for miR-142-5p (assay ID: 002248), miR-590-5p (assay ID: 001984), miR-301a (assay ID: 000528), miR-503 (assay ID: 001048), RNU6 (assay ID: 001973), miR-125b (assay ID: 000449) and miR-374b (assay ID: 001319) starting with 10 ng of total RNA, on an ABI Prism 7900 HT. miR-374b was chosen as an endogenous control for PBMC samples because it was one of the miRNA that correlated well with the median Cq of all miRNA (r = 0,94), it had a low variance and was well expressed (mean Cq = 18.0) in the card assays. RNU6 was chosen as an endogenous control for other sample sources. Relative expression between a sample and a reference was calculated according to the 2^−ΔΔCq^ method (PE Applied Biosystems 1997).

### Gene expression microarray analysis

Gene expression data for PBMC from 12 CAMR and 12 STA using Affymetrix Human Genome U133 Plus 2.0 arrays (Affymetrix, Santa Clara, CA, USA) were obtained from the study of Lozano *et al.* [17]. We then selected probes corresponding to genes predicted as being targeted by miR-142-5p according to the miRDB database [18] (*e.g.* 887 genes).

### Statistical analysis

Receiver operating characteristic (ROC) analysis, non-parametric Mann–Whitney tests or Kruskal Wallis with Dunn's ad hoc tests were used for group comparisons with the Graph PadPrism v.4 software. Differences were defined as statistically significant when p<0.05 (*), p<0.01 (**).

## Results

### CAMR is associated with a 10 miRNA blood signature

**Figure 1:**
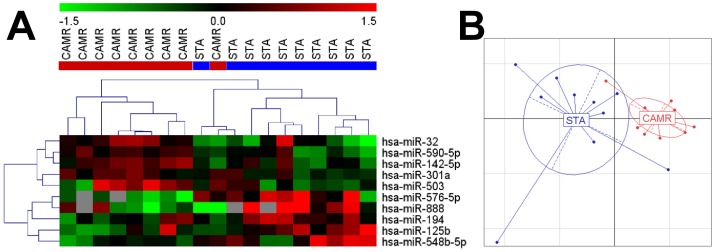
Unsupervised clustering (A) and PCA (B) of STA and CAMR groups with the 10 selected miRNAs. The heatmap represents normalized and color-coded relative expression values (2^−ΔΔCq^) in which red values indicate over-expression and green values indicate under-expression. The PCA graph represents the first (x axis) and second (y axis) components of the PCA analysis.

Using Taqman low density arrays (TLDA), the expression of 381 miRNAs was measured in PBMC from 9 CAMR and 10 STA patients. 257 miRNAs were expressed with a cycle of quantification (Cq) below 35 in at least half of the samples in each group. Among them, we selected the 10-top ranked miRNAs according to Mann–Whitney tests between the two groups of patients (Table 1). Half of the miRNAs were over-expressed (miR-301a, miR-590-5p, miR-142-5p, miR-32 and miR-503) and half were under-expressed (miR-888, miR-576-5p, miR-548b-5p, miR-125b and miR-194) in the blood of patients with CAMR compared to STA. Using an unsupervised clustering analysis and a principal component analysis (PCA) the two patient groups could be distinguished based on the expression of these 10 miRNAs (Figure 1).

**Table 1:** Ten selected miRNAs associated with CAMR.

	miRNA name	Fold Change (log_2_CAMR/STA)	Mann-Whitney p-value	Cq mean
1	hsa-miR-301a	0.42	0.002	26.4
2	hsa-miR-576-5p	−2.18	0.005	20.1
3	hsa-miR-590-5p	0.49	0.010	26.6
4	hsa-miR-125b	−0.73	0.013	21.6
5	hsa-miR-32	0.59	0.013	31.7
6	hsa-miR-142-5p	0.55	0.017	27.3
7	hsa-miR-888	−1.61	0.021	15.8
8	hsa-miR-548b-5p	−0.88	0.022	21.3
9	hsa-miR-194	−0.57	0.022	23.1
10	hsa-miR-503	0.93	0.022	33.1

Expression fold changes (in log_2_) of CAMR related to STA, p-values from Mann-Whitney tests with 2^−ΔΔCq^ values and Cq means from TLDA are displayed.

### miR-142-5p is a specific biomarker of CAMR

**Figure 2:**
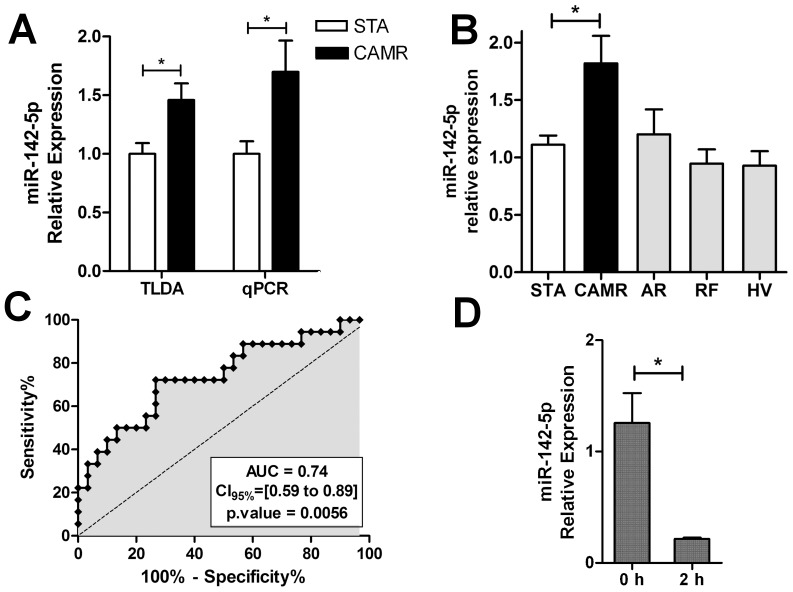
miR-142-5p as a blood biomarker of CAMR A) TLDA and individual qPCR measurements performed in same PBMC samples (10 STA and 9 CAMR) are displayed for miR-142-5p. B) miR-142-5p over-expression in CAMR compared to STA only was validated on additional PBMC samples (30 STA and 18 CAMR; p = 0.0058). The expression of miR-142-5p was measured in the blood of patients with different clinical statuses (9AR, 10RF and 8HV). Non parametric Dunn's ad hoc test was used to compare all groups to STA. C) ROC curve analysis of qPCR data for these validation samples (30 STA and 18 CAMR) revealed an area under the curve (AUC) of 0.74 (CI_95%_ = [0.59 to 0.89]; p = 0.0056). D) miR-142-5p expression was rapidly decreased in the PBMC of 3 HV after PHA (2 µg/mL) and Il-2 (150 U/mL) stimulation. Mean ±SEM miR-142-5p expression relative to miR-374b is represented.

We focused on miR-142-5p because of its specificity for the hematopoietic cell lineage [19], [20] and its increased expression in the blood of patients with CAMR. We first confirmed its significant over-expression using qPCR assays on the same PBMC samples used for profiling (p = 0.043) (Figure 2A). We also measured the expression of four miRNAs from the signature: miR-301a, miR-125b, miR-503 and miR-590-5p (Figure S1). miR-590-5p was significantly up-regulated in blood from patients with CAMR according TLDA and PCR (p = 0.049). miR-125b and miR-503 displays down- and up-regulation, respectively, without reaching significance (p-value>0.05) whereas no difference was observed for miR-301a. We then validated the over-expression of miR-142-5p on an independent group of samples composed of PBMC from 18 CAMR compared to 30 STA patients (p = 0.0058). The ROC analysis performed on these validation samples indicated that miR-142-5p discriminated well CAMR and STA patients with an area under the curve (AUC) of 0.74 (CI_95%_ = [0.59 to 0.89]; p = 0.0056) (Figure 2C). In order to investigate the biomarker capacity of circulating miR-142-5p, this miRNA was measured by qPCR in plasma from 10 CAMR and 10 STA. No difference was observed between the two groups of patients (Figure S2).

To assess its specificity as a biomarker of CAMR, we measured its expression in PBMC from patients with acute rejection (AR, n = 9), patients with renal failure exhibiting deteriorating kidney function for non-immunological causes without renal transplantation (RF, n = 10) and healthy volunteers (HV, n = 8) (Figure 2B). Comparing to STA, the expression of miR-142-5p remained significantly over-expressed in the blood of CAMR patients only (p<0.05). No significant differential expression was observed with HV, suggesting no relationship between miR-142-5p and the immunosuppressive treatment (Figure 2B). ROC analyses indicated that miR-142-5p discriminated CAMR from RF patients very well (AUC = 0.82, CI95% = [0.64 to 0.99]; p = 0.0063) and less efficiently CAMR from AR patients (AUC = 0.72, CI95% = [0.50 to 0.94]; p = 0.064) (Figure S3). Finally, the expression of miR-142-5p was not significantly increased in the blood of patients with AR compared to STA (Figure 2B), and was significantly decreased in PBMC from HV after 2 hours stimulation with phytohemagglutinin A (PHA; 2 µg/mL) and Interleukin 2 (IL-2; 10 ng/mL) (p = 0.017), suggesting that its over-expression in CAMR was not due to non-specific cell activation (Figure 2D).

Altogether, these data show that the expression of miR-142-5p was specifically and significantly increased in the blood of patients with CAMR but not in the blood of patients with other types of renal dysfunction. In addition, miR-142-5p expression did not appear to be influenced by immunosuppressive treatment and the increase in CAMR did not seem to be the result of non-specific activation.

### miR-142-5p is increased in kidney grafts displaying histological signs of CAMR

**Figure 3:**
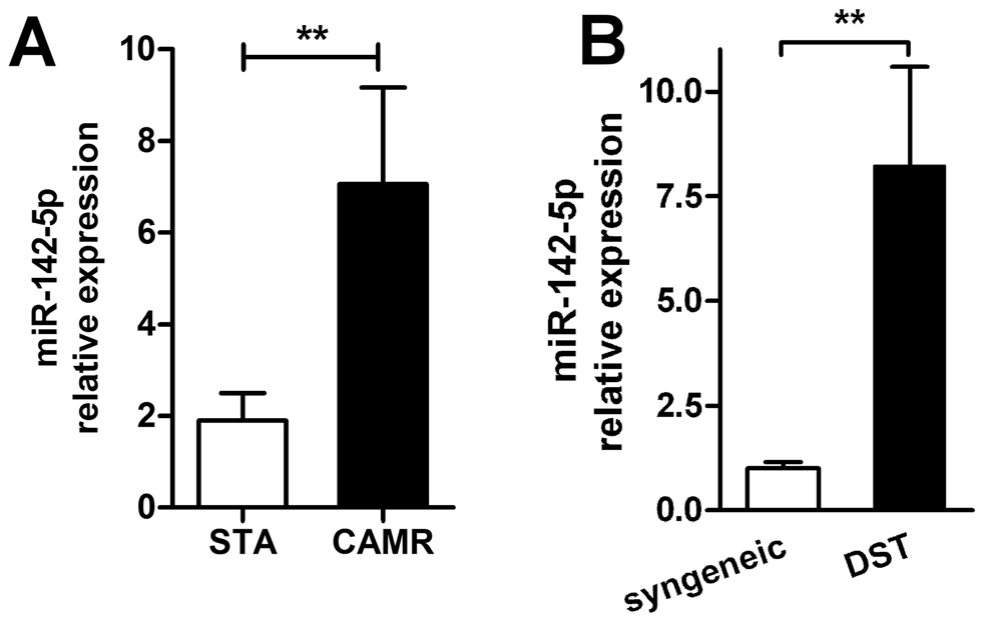
miR-142-5p expression in the graft A) miR-142-5p expression was increased in the biopsies of patients with CAMR lesions compared to STA patients with no histological lesions of CAMR (20 CAMR, 18 STA, p = 0.0082); B) miR-142-5p expression was increased in the grafts of DST-treated rats compared to untreated syngeneic controls 100 days after transplantation (5 DST, 5 syngeneic, p = 0.0079). Mean ±SEM miR-142-5p expression relative to RNU6 is represented.

miR-142-5p was measured by qPCR in kidney graft biopsies from 21 patients with CAMR and 18 patients with a well-functioning graft and no histological lesions of CAMR (STA). An increased expression of miR-142-5p was observed in biopsies with CAMR compared to biopsies from STA (p = 0.0082; Figure 3A). ROC analysis displayed a good discrimination of both population with miR-142-5p expression (AUC = 0.75, CI_95%_ = [0.60 to 0.91]; p = 0.0078). No correlation was found between the expression in PBMC and in biopsy harvested at the same time in a subset of 6 patients with CAMR (Spearman r = −0.32, p = 0.53) (Figure S4). In parallel, we analyzed the expression of miR-142-5p in a rat model of CAMR where rat recipients of MHC-mismatched cardiac allografts received 2 pre-graft donor-specific transfusions (DST). 100 days after transplantation, the grafts displayed histological signs of chronic antibody mediated rejection, with C4d deposit, comparable to what is observed in human graft biopsies [14]. Similar to the findings in the human biopsies, 100 days after transplantation, miR-142-5p expression was increased in the rat grafts displaying signs of CAMR compared to syngeneic grafts (p = 0.0079; Figure 3B).

### CAMR harbored a fingerprint of miR-142-5p target genes

**Figure 4:**
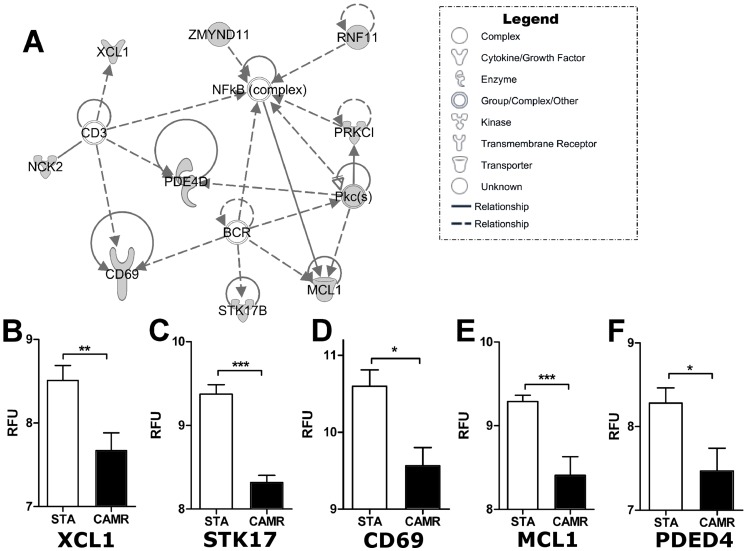
Gene network composed of immune-related genes potentially targeted by miR-142-5p A) This gene network was built with IPA software and down-regulated potential targets are highlighted in grey. Corresponding gene expression based on microarrays data, in relative fluorescent units (RFU), are displayed for XCL1 (B), STK17 (C), CD69 (D), MCL1 (E) and PDE4D (F) (CAMR = 12 and STA = 12).

Using the online database miRDB (http://mirdb.org), we identified 887 genes predicted as being potentially targeted by miR-142-5p [18]. Among them, 595 genes were expressed in the microarray dataset previously obtained by analyzing patients with a similar clinical profile [17]. The over-expression of miR-142-5p in patients with CAMR correlated with the significant down-regulation of 41 genes (SAM q-value<10%, Fold Change_CAMR/STA_<1) reported in Table S2. Interestingly, the Ingenuity Pathways Analysis (IPA) performed on these 41 genes indicated a major gene network related to “cell-mediated immune response” including links with the NFκB complex, and T- and B-cell receptors (TCR and BCR) (Figure 4A). Figure 4 (B to F) illustrates some of these genes that play important roles in leukocyte functions: CD69, PDE4D (phosphodiesterase 4D, cAMP-specific), XCL1 (chemokine (C motif) ligand 1), STK17B (serine/threonine kinase 17b) and MCL1 (myeloid cell leukemia sequence 1) [21]–[24]

## Discussion

More than 60% of human coding genes may be under the control of miRNAs [25] and growing evidence indicates that they play important roles in innate and adaptive immune functions [26]. The dynamic expression of ubiquitous and hematopoietic-specific miRNAs follows developmental and activation states of immune cells [20], [27], [28]. Consequently, miRNA expression deregulation may have dramatic effects. They have been shown to be involved in human pathology and their expression has also been reported in renal transplant patients undergoing acute cellular rejection [7]–[9], fibrosis [10], [11] and operational tolerance [12].

Because of their fine-tuned modulation, miRNAs may also serve as markers for pathological patterns involving a high number of molecular interactions. Herein, in the search for minimally invasive biomarkers of CAMR, we measured the expression of a large panel of miRNAs in PBMC of patients with established CAMR compared to patients with stable graft function. Using non-parametric supervised analysis, we selected a short-list of 10 top-ranked miRNAs expressed in the blood of patients with CAMR and we focused on the expression of miR-142-5p, a specific hematopoietic cell lineage miRNA [19], [20]. Its over-expression was confirmed on the same group of patients and validated in an independent group of patients with comparable clinical criteria. miR-142-5p was increased in the blood and biopsies of patients with CAMR but not in the blood of patients with renal failure, suggesting that its over-expression is associated with immunological disorders rather than renal dysfunction. Interestingly, an increase expression of miR-142-5p has also been reported in biopsies from 14 patients with interstitial fibrosis and tubular atrophy including 5 patients exhibiting lesions of CAMR [11]. Such increased expression of miR-142-5p was also observed in cardiac transplant biopsies from rats displaying lesions of chronic antibody-mediated rejection [14]. In this model, typical histological lesions with intragraft C4d deposits and anti-donor circulating antibodies are observed in a similar way as in humans [14]. This increased intragraft expression of miR-142-5p in a rodent cardiac allotransplantation model [14] suggests that this miRNA could be used to diagnose humoral rejection in organs other than the kidney; that needs to be confirm in further studies in humans. Furthermore, miR-142-5p expression was decreased in PHA-activated PBMC and was not increased in the blood of patients with acute rejection, excluding the possibility of non-specific T cell activation in the blood and biopsies of patients with CAMR. The absence of modulation of this miRNA in the blood of patients under immunosuppression (STA and AR) compared to healthy volunteers without immunosuppressive treatment highly suggests that its expression is not influenced by the treatment and indicates miR-142-5p could be a blood biomarker specific for CAMR. Finally, ROC analyses indicated that miR-142-5p discriminated well CAMR from STA or RF patients. Of course, only a prospective study, with large number of samples, would establish the blood diagnostic properties of miR-142-5p compared to clinical parameters such as creatinemia.

miR142-5p has been shown to be over-expressed in other pathological contexts, such as cancers [29] and immunologically-related disorders [30], small bowel inflammation [31] but also in renal fibrosis where inflammation occurs [11], [32] and in biopsies from renal transplant patients with AR [7]. Moreover, Anglicheau and colleagues demonstrated miR-142-5p expression in biopsies from patients with AR is directly correlated to the infiltrate [7]. Considering that miR-142-5p is enriched in the hematopoietic cell lineage [19], [20], we speculate that this miRNA may be carried into the graft by leukocytes, to counteract local inflammatory process. This is in line with the fact that the allograft itself can induce regulatory processes to prevent rejection, and that these processes still persist when rejection occurs, likely due to the persistence of exposure to alloantigens [33]. We speculated that the observed discrepancy in miR-142-5p expression between AR and CAMR patients, *i.e.* the absence of increased expression of miR-142-5p in PBMC from patients with AR in opposition to CAMR whereas its expression was previously found increased in AR biopsies [7], may arise from the difference in kinetics of AR episodes and CAMR. Finally, because miR-142-5p is expressed by hematopoietic cells, a difference in PBMC would appear only if regulation mechanisms stemming from this miRNA are involved. This possible regulatory role of miR-142-5p in inflammatory processes is reinforced by its inhibitory effect on growth on lung cancer cell lines [34]. We identified 41 genes that were down-regulated in a microarray dataset obtained in our laboratory on PBMC from patients in similar clinical situations (CAMR and STA) [17], [18] and that were predicted as miR-142-5p targets by the miRDB. Moreover, we reported that this miRNA is involved in different cell network pathways and among them an immune-related network pathway (Figure 4), showing that a clear signature of miR-142-5p target genes is present in the blood and graft from patients with CAMR [22]. In addition, recent findings demonstrated the over-expression of miR-142-5p in CD4^+^ T lymphocytes from patients with systemic lupus erythematous reduced autoimmune activity and IgG production whereas its inhibition in HV CD4^+^ T cells induced T cell over-activation and B cell hyperstimulation [35]. Despites functional experiments are required with PBMC from patients with CAMR, these data suggest that miR-142-5p may play a regulatory role in the immune response, possibly involved in controlling the leukocyte activation.

Among the 10 top-ranked miRNAs identified in the TLDA assays, miRNAs other than miR-142-5p were modulated that have been reported as involved in immunity and renal function. The decreased expression of miR-125b in patients with CAMR is in accordance with the fact that low expression of miR-125b is required for the acquisition of effector T cell functions [36]. This profile fits with our previously published data reporting on the over-expression of some molecules such as TBX21, GRZB and TLR4 in the blood and biopsies of patients with CAMR [15], [37], [38]. Similarly, miR-194 was previously reported in renal ischemia reperfusion injury [39] and fibrosis [32].

Finally, these data suggest that PBMC may be an adequate compartment for identification of miRNAs as biomarkers in transplantation. Our data show that miR-142-5p appears to be a promising biomarker for CAMR diagnosis either in PBMC or biopsies. Validation in larger cohorts of patients should establish its diagnostic power as a biomarker for CAMR.

## Supporting Information

Figure S1 TLDA and individual qPCR assays for four miRNAs. TLDA and individual qPCR measures performed in same PBMC samples (10 STA, 9 CAMR) are displayed for miR-125b (A), miR-301a (B), miR-503 (C) and miR-590-5p. Mean ± SEM of relative expression (2^−ΔΔCq^ relative to miR-374b) are represented.
(TIFF)

Figure S2 Measure of miR-142-5p expression in plasma from 10 CAMR and 10 STA (p = 0.912). Mean ± SEM of relative expression (2^−ΔΔCq^ relative to RNU6) are represented.
(TIFF)

Figure S3 ROC curve of miR-142-5p expression in CAMR compared to 9AR (A) and 10RF (B) ROC curve analyses were performed on the expression of miR-142-5p with the validation samples (18 CAMR).
(TIFF)

Figure S4 Measure of miR-142-5p expression in PBMC and biopsy harvested at the same time from 6 CAMR.
(TIFF)

Table S1 Detailed clinical data. The daily proteinuria above 0.5 g/d (i.e.: 0.58 g/d) that was observed for patients STA-11 and STA-14 was not confirmed on previous dosages and afterward until now, thus those patients were considered highly stable.na: non attribuable; nd: non determined; K: kidney; P: pancreas; CSA: cyclosporine A; FK: Tacrolimus; CS: Corticosteroid; My: Mycophenolate;BA: Basiliximab; IVIG: Intravenous immunoglobulin; inh-mTor: mTor inhibitor; AZA: Azathioprine, CNI: Calcineurin inhibitor; sCAMR: suspicious CAMR.
(DOC)

Table S2 Down-expressed genes in CAMR compared to STA (SAM q-value<10%, Fold Change _CAMR/STA_<1) and predicted as targets for miR-142-5p by miRDB [2], [3].
(DOC)

Methods S1 Expanded description of methods.
(DOC)
