# Supplementary figures and images for: Expression of miR-142-5p in Peripheral Blood Mononuclear Cells from Renal Transplant Patients with Chronic Antibody-Mediated Rejection
Source: PLoS One. 2013 Apr 5;8(4):e60702. doi: 10.1371/journal.pone.0060702 (PMC3618046; doi:10.1371/journal.pone.0060702)

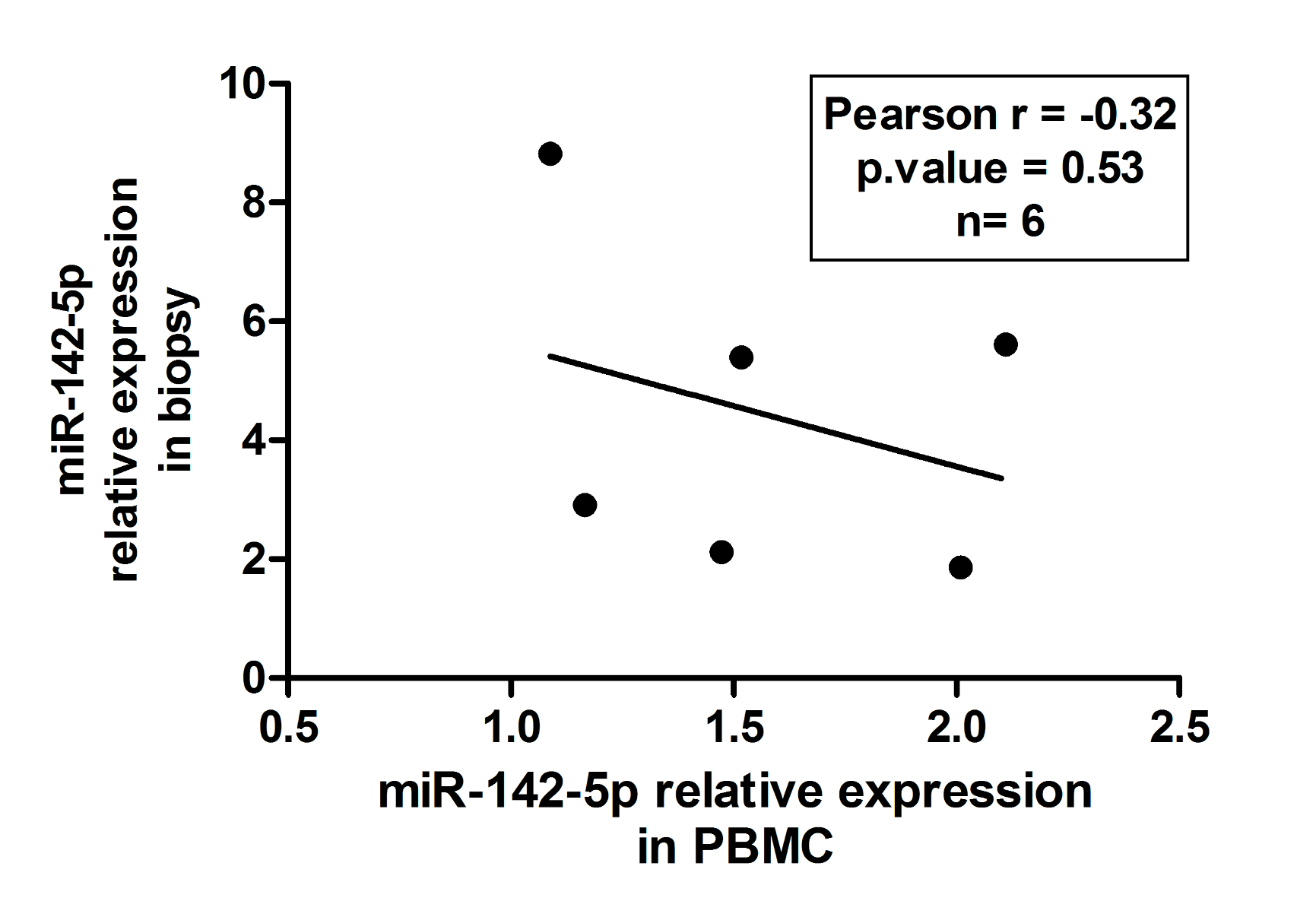

Supplement: Figure S4 — Measure of miR-142-5p expression in PBMC and biopsy harvested at the same time from 6 CAMR. (TIFF) [file pone.0060702.s004.tif]
