# Supplementary material for: Expression of miR-142-5p in Peripheral Blood Mononuclear Cells from Renal Transplant Patients with Chronic Antibody-Mediated Rejection
Source: PLoS One. 2013 Apr 5;8(4):e60702. doi: 10.1371/journal.pone.0060702 (PMC3618046; doi:10.1371/journal.pone.0060702)
Supplement: Table S1 — Detailed clinical data. The daily proteinuria above 0.5 g/d (i.e.: 0.58 g/d) that was observed for patients STA-11 and STA-14 was not confirmed on previous dosages and afterward until now, thus those patients were considered highly stable.na: non attribuable; nd: non determined; K: kidney; P: pancreas; CSA: cyclosporine A; FK: Tacrolimus; CS: Corticosteroid; My: Mycophenolate;BA: Basiliximab; IVIG: Intravenous immunoglobulin; inh-mTor: mTor inhibitor; AZA: Azathioprine, CNI: Calcineurin inhibitor; sCAMR: suspicious CAMR. (DOC) [file pone.0060702.s005.doc]

1/3

2/3

3/3
