# Supplementary material for: Expression of miR-142-5p in Peripheral Blood Mononuclear Cells from Renal Transplant Patients with Chronic Antibody-Mediated Rejection
Source: PLoS One. 2013 Apr 5;8(4):e60702. doi: 10.1371/journal.pone.0060702 (PMC3618046; doi:10.1371/journal.pone.0060702)
Supplement: Table S2 — Down-expressed genes in CAMR compared to STA (SAM q-value<10%, Fold Change CAMR/STA<1) and predicted as targets for miR-142-5p by miRDB [2], [3]. (DOC) [file pone.0060702.s006.doc]

| **Symbol** | **Name** | **Probe ID** | **FC (unlogged)** | **q-value (%)** |
| --- | --- | --- | --- | --- |
| CD69 | CD69 molecule | 209795_at | 0.09 | 2.68 |
| STK17B | serine/threonine kinase 17b | 243797_at | 0.09 | 0.00 |
| XCL1 | chemokine (C motif) ligand 1 | 206366_x_at | 0.11 | 3.76 |
| CLDND1 | claudin domain containing 1 | 208925_at | 0.27 | 0.00 |
| B4GALT6 | UDP-Gal:betaGlcNAc beta 1,4- galactosyltransferase, polypeptide 6 | 235333_at | 0.32 | 4.17 |
| MCL1 | myeloid cell leukemia sequence 1 | 200796_s_at | 0.34 | 0.00 |
| ZEB2 | zinc finger E-box binding homeobox 2 | 233031_at | 0.35 | 4.17 |
| ZMYND11 | zinc finger, MYND domain containing 11 | 202137_s_at | 0.35 | 0.00 |
| TPR | translocated promoter region (to activated MET oncogene) | 1557227_s_at | 0.35 | 0.00 |
| REV3L | REV3-like, catalytic subunit of DNA polymerase zeta (yeast) | 238736_at | 0.36 | 0.00 |
| MARCH6 | membrane-associated ring finger (C3HC4) 6 | 201737_s_at | 0.37 | 0.00 |
| ZBTB38 | zinc finger and BTB domain containing 38 | 236557_at | 0.38 | 4.17 |
| ZBTB38 | zinc finger and BTB domain containing 38 | 1558733_at | 0.38 | 6.46 |
| PTPN4 | protein tyrosine phosphatase, non-receptor type 4 (megakaryocyte) | 205171_at | 0.39 | 3.76 |
| COPA | coatomer protein complex, subunit alpha | 214336_s_at | 0.40 | 0.00 |
| ARID2 | AT rich interactive domain 2 (ARID, RFX-like) | 231090_s_at | 0.43 | 4.17 |
| ZBTB11 | zinc finger and BTB domain containing 11 | 242433_at | 0.43 | 3.76 |
| FAM63B | family with sequence similarity 63, member B | 214691_x_at | 0.44 | 2.68 |
| ZNF652 | zinc finger protein 652 | 205594_at | 0.45 | 2.68 |
| PITPNC1 | phosphatidylinositol transfer protein, cytoplasmic 1 | 219155_at | 0.45 | 7.79 |
| ZMYND11 | zinc finger, MYND domain containing 11 | 1554159_a_at | 0.45 | 4.17 |
| PDK1 | pyruvate dehydrogenase kinase, isozyme 1 | 206686_at | 0.46 | 2.83 |
| UHMK1 | U2AF homology motif (UHM) kinase 1 | 1552656_s_at | 0.46 | 2.83 |
| NCK2 | NCK adaptor protein 2 | 203315_at | 0.48 | 3.76 |
| EID1 | EP300 interacting inhibitor of differentiation 1 | 211698_at | 0.49 | 7.36 |
| ATG16L1 | ATG16 autophagy related 16-like 1 (S. cerevisiae) | 220521_s_at | 0.50 | 7.59 |
| MAGT1 | magnesium transporter 1 | 221553_at | 0.50 | 2.83 |
| SETD2 | SET domain containing 2 | 220946_s_at | 0.52 | 8.57 |
| PRKCI | protein kinase C, iota | 209678_s_at | 0.52 | 9.91 |
| LARS | leucyl-tRNA synthetase | 223888_s_at | 0.53 | 3.76 |
| HIPK1 | homeodomain interacting protein kinase 1 | 1552516_a_at | 0.54 | 9.91 |
| TLE4 | transducin-like enhancer of split 4 (E(sp1) homolog, Drosophila) | 235765_at | 0.55 | 4.17 |
| ZNF587 | zinc finger protein 587 | 231820_x_at | 0.55 | 4.17 |
|  |  |  |  |  |
| FGFR1OP2 | FGFR1 oncogene partner 2 | 223263_s_at | 0.56 | 4.17 |
| PITPNC1 | phosphatidylinositol transfer protein, cytoplasmic 1 | 1568949_at | 0.56 | 4.17 |
| HELQ | helicase, POLQ-like | 1554341_a_at | 0.57 | 5.97 |
| PAPOLA | poly(A) polymerase alpha | 228569_at | 0.57 | 3.76 |
| PDE4D | phosphodiesterase 4D, cAMP-specific | 204491_at | 0.57 | 6.46 |
| ZCCHC11 | zinc finger, CCHC domain containing 11 | 217594_at | 0.58 | 3.63 |
| SNAP23 | synaptosomal-associated protein, 23kDa | 209131_s_at | 0.59 | 7.79 |
| KIAA0494 | KIAA0494 | 201777_s_at | 0.60 | 7.79 |
| TLE4 | transducin-like enhancer of split 4 (E(sp1) homolog, Drosophila) | 233575_s_at | 0.60 | 8.12 |
| EID1 | EP300 interacting inhibitor of differentiation 1 | 208669_s_at | 0.60 | 4.17 |
| TLE4 | transducin-like enhancer of split 4 (E(sp1) homolog, Drosophila) | 216997_x_at | 0.61 | 6.46 |
| LARS | leucyl-tRNA synthetase | 217810_x_at | 0.65 | 9.91 |
| DENR | density-regulated protein | 234915_s_at | 0.65 | 9.91 |
| SH2D1A | SH2 domain protein 1A | 211209_x_at | 0.66 | 9.91 |
| RNF11 | ring finger protein 11 | 208924_at | 0.66 | 7.79 |
| SH2D1A | SH2 domain protein 1A | 211211_x_at | 0.67 | 7.59 |
