# Supplementary material for: Expression of miR-142-5p in Peripheral Blood Mononuclear Cells from Renal Transplant Patients with Chronic Antibody-Mediated Rejection
Source: PLoS One. 2013 Apr 5;8(4):e60702. doi: 10.1371/journal.pone.0060702 (PMC3618046; doi:10.1371/journal.pone.0060702)
Supplement: Methods S1 — Expanded description of methods. (DOC) [file pone.0060702.s007.doc]

**Supplementary Methods S1**

**Sample preparation:** Renal graft biopsies were collected using 18-G disposable needles, immediately snap-frozen in liquid nitrogen and stored at -80°C. Total RNA was extracted from frozen biopsies with a polytron homogeneizer in TRIzol® reagent (Invitrogen, Cergy Pontoise, France) and using the TRIzol® method according to the manufacturer’s instructions.

Venous blood samples were collected in EDTA vacutainers and processed for analysis within 4 hours. Plasma samples were obtained after centrifugation (2,000g for 15minutes) and frozen at -80°C. RNA was extracted with 300µL of plasma and 1000µL of TRIzol® reagent. Peripheral Blood Mononuclear Cells (PBMC) were separated on a Ficoll layer (Eurobio, Les Ulis, France) and frozen in TRIzol® reagent (Invitrogen, Cergy Pontoise, France) at -80°C. RNA was extracted from peripheral blood using the TRIzol® method. RNA quality and quantity were determined using an Agilent 2100 BioAnalyzer (Palo Alto, CA, USA) and a Nanodrop (Labtech, Palaiseau, France) respectively.

**PBMC activation and analysis:** PBMC from 3 HV (2×10^6^ cells/well) were cultured in 6 wells plate with phytohemagglutinin A (PHA, 2µg/mL, Sigma-Merck, Darmstadt, Germany) and Il-2 (150U/mL) in RPMI 1640 medium supplemented with penicillin (100 U/ml), streptomycin (100 μg/ml), sodium pyruvate (1 mM), and 10% heat-inactivated fetal calf serum (FCS) (Gibco, Cergy-Pontoise, France). Two hours after stimulations, cells were frozen in TRIzol® reagent (Invitrogen, Cergy Pontoise, France) at -80°C.

**microRNA profiling:** miRNA profiling was performed using TaqMan Arrays MicroRNA Cards pool A set v2.0 (Applied Biosystems, Foster City, CA, USA) with pre-amplification following manufacturer’s recommendations. Briefly, 200ng of total RNA was reverse transcribed using the Megaplex RT stem-loop primer pool A set v2.0 and RT product was pre-amplified by 12 cycles of PCR reaction using Megaplex PreAmp Primers pool A. Quantitative PCR reaction was performed with pre-amplified cDNA and with TaqMan Array MicroRNA Cards pool A set v2.0 on an ABI Prism 7900 HT (Applied Biosystems) with SDS software version 2.3. Cq were obtained from RQ Manager v.1.2.1 (Applied Biosystems). For further data analysis, miRNAs with more than half cycle of quantification (Cq) values above 35 per group were removed. For each sample, normalization was performed by subtraction of the median of measured Cq, resulting in the ∆Cq value . Because median is less sensitive to extreme values than mean, recommended by Mestdagh *et al.*, we used median value. The 2^−ΔΔCq^ value, corresponding to the relative expression, was calculated using the median of ∆Cq values from STA group as an artificial calibrator sample. In order to identify potential differential miRNAs, miRNAs were ranked using p-values from non-parametric Mann-Whitney tests which does not require normal assumptions and asymptotic conditions. These p-values are uncorrected for multiple testing and thus are not an absolute identification of significant differential miRNAs, further validation are required for each miRNA.

**microRNA individual assays:** Individual microRNA expression was measured with the TaqMan miRNA assay protocol (Applied Biosystems, Foster City, CA, USA) using probes for miR-142-5p (assay ID: 002248), miR-590-5p (assay ID: 001984), miR-301a (assay ID: 000528), miR-503 (assay ID: 001048) RNU6 (assay ID: 001973), miR-125b (assay ID: 000449) and miR-374b (assay ID: 001319) starting with 10ng of total RNA, on an ABI Prism 7900 HT. miR-374b was chosen as an endogenous control for PBMC samples because it was one of the most correlated miRNA to the median Cq all miRNA (r=0,94) with a low variance and well expressed (mean Cq=18.0) in the card assays. RNU6 was chosen as an endogenous control for other sample sources. Relative expression between a sample and a reference was calculated according to the 2^−ΔΔCq^ method (PE Applied Biosystems 1997).

**Gene expression microarrays analysis:** Gene expression data from PBMC from 12 CAMR and 12 STA on Affymetrix Human Genome U133 Plus 2.0 arrays (Affymetrix, Santa Clara, CA, USA) were obtained from the study of Lozano *et al.* (Lozano*, et al.* 2011a). Then, we selected probes corresponding to genes predicted as targeted by miR-142-5p by the miRDB database (Wang 2008) (*e.g.* 887 genes). Among the 1,342 selected probes, unique genes corresponding to probes which exhibited a q-value inferior to 10% in a SAM test with 1,000 permutations and a decrease expression in the CAMR compared to STA were analyzed using Ingenuity Pathays Analysis (IPA) software.

**Statistical analysis:** Receiver operating characteristic (ROC) analysis, non-parametric Mann–Whitney tests or Kruskal Wallis with Dunn’s ad hoc tests were used for group comparisons with the Graph PadPrism v.4 software. Differences were defined as statistically significant when p<0.05 (*), p<0.01 (**).

**SI references**

1. Mestdagh P, Van Vlierberghe P, De Weer A, Muth D, Westermann F, et al. (2009) A novel and universal method for microRNA RT-qPCR data normalization. Genome Biol 10: R64.

2. Lozano JJ, Pallier A, Martinez-Llordella M, Danger R, Lopez M, et al. (2011) Comparison of transcriptional and blood cell-phenotypic markers between operationally tolerant liver and kidney recipients. Am J Transplant 11: 1916-1926.

3. Wang X (2008) miRDB: a microRNA target prediction and functional annotation database with a wiki interface. RNA 14: 1012-1017.
